# Cationic Thiolated Poly(aspartamide) Polymer as a Potential Excipient for Artificial Tear Formulations

**DOI:** 10.1155/2016/2647264

**Published:** 2016-05-23

**Authors:** Mária Budai-Szűcs, Gabriella Horvát, Barnabás Áron Szilágyi, Benjámin Gyarmati, András Szilágyi, Szilvia Berkó, Piroska Szabó-Révész, Giuseppina Sandri, Maria Cristina Bonferoni, Carla Caramella, Judit Soós, Andrea Facskó, Erzsébet Csányi

**Affiliations:** ^1^Department of Pharmaceutical Technology, Faculty of Pharmacy, University of Szeged, Eötvös utca 6, Szeged 6720, Hungary; ^2^Soft Matters Group, Department of Physical Chemistry and Materials Science, Budapest University of Technology and Economics, Budafoki út 8, Budapest 1111, Hungary; ^3^Department of Drug Sciences, Faculty of Pharmacy, University of Pavia, Viale Taramelli 12, 27100 Pavia, Italy; ^4^Department of Ophthalmology, Faculty of Medicine, University of Szeged, Korányi fasor 10-11, Szeged 6720, Hungary

## Abstract

Dry eye disease is a relatively common ocular problem, which causes eye discomfort and visual disorders leading to a decrease in the quality of life. The aim of this study was to find a possible excipient for eye drop formulations, which is able to stabilize the tear film. A cationic thiolated polyaspartamide polymer, poly[(*N*-mercaptoethylaspartamide)-co-(*N*-(*N*′,*N*′-dimethylaminoethyl)aspartamide)] (ThioPASP-DME), was used as a potential vehicle. Besides satisfying the basic requirements, the chemical structure of ThioPASP-DME is similar to those of ocular mucins as it is a protein-like polymer bearing a considerable number of thiol groups. The solution of the polymer is therefore able to mimic the physiological properties of the mucins and it can interact with the mucus layer via disulphide bond formation. The resultant mucoadhesion provides a prolonged residence time and ensures protective effect for the corneal/conjunctival epithelium. ThioPASP-DME also has an antioxidant effect due to the presence of the thiol groups. The applicability of ThioPASP-DME as a potential excipient in eye drops was determined by means of ocular compatibility tests and through examinations of the interactions with the mucosal surface. The results indicate that ThioPASP-DME can serve as a potential eye drop excipient for the therapy of dry eye disease.

## 1. Introduction

Dry eye disease (DED) has been reported to afflict 7–33% of the population, thereby reducing their quality of life. For normal vision, continuous moistening of the ocular surface is needed. Important roles are played in this by a sufficient quality of tears, maintenance of the normal composition of the tear film, normal lid closure, and regular blinking [1, 2]. If equilibrium is lost, the DED can occur, resulting in eye discomfort and visual disturbance [2, 3].

DED is accompanied by changes in mucin distribution and glycosylation, a dysfunction of MUC4 and MUC5AC and a high calcium level [4]. The mucins act as a lubricant during blinking, stabilize the preocular tear film to prevent desiccation of the epithelium, and form a barrier against pathogen penetrance [5]. Intracellular calcium is responsible for cationic shielding to keep negatively charged mucins condensed and packed within the granules of goblet cells. In the event of enhanced calcium release, the granules swell, become detached from the cell surface, form large aggregates, and diffuse onto the epithelial surface. This leads to a lower degree of hydration of the mucus coverage, which contains dry spots, resulting in decreased tear film stability [4].

One way to stabilize the tear film in cases of DED is to use liquid thiolated polymer formulations, whose structures are similar to those of ocular mucins, as they are protein-like polymers bearing a considerable number of thiol groups. The solutions of such polymers are therefore able to mimic the physiological properties of mucins, such as tear film stabilization. The formation of disulphide bonds with the mucus layer leads to strong mucoadhesion, which may be further strengthened by the formation of ionic bonds between the cationic groups of the excipient and the anionic groups of the mucins. The strong adhesion promotes a prolonged residence time and a protective effect for the corneal/conjunctival epithelium. Liquid formulations also serve as lubricants, prolonging the breakup time of the tear film. Moreover, thiolated polymers have antioxidant and radical scavenging properties and can therefore be useful excipients in artificial tear formulations for the therapy of DED [4, 6].

We earlier described thiolated poly(aspartic acid) (ThioPASP) polymers, which are biocompatible [7, 8],* in situ* gelling, and potential ophthalmic vehicles [9, 10]. The aims of the present study were to synthetize and characterize a cationic thiolated poly(aspartamide) bearing both cationic tertiary amine and redox-responsive thiol pendant groups as a potential mucoadhesive and tear film-stabilizing excipient in the therapy of DED. Ocular compatibility tests were performed to determine its applicability as a potential excipient in eye drops.

## 2. Materials and Methods

### 2.1. Materials

For the synthesis of the polymers, L-aspartic acid (Merck, extra pure), phosphoric acid (Sigma Aldrich, 99%), cysteamine (Acros Organics, 95%),* N*,*N*-dimethylethylenediamine (Sigma Aldrich, 95%), ethyl acetate (Reanal Hungary, a.r.), acetone (Reanal Hungary, a.r.), and* N*,*N*-dimethylformamide (DMF) were used without further purification. To mimic the oxidative effect on the ocular surface, 20% w/w 1 M NaBrO_3_ was used as model oxidant in the formulations. A phosphate-buffered saline (PBS) solution of pH = 7.4 was prepared by dissolving 8 g dm^−3^ NaCl, 0.2 g dm^−3^ KCl, 1.44 g dm^−3^ Na_2_HPO_4_·2H_2_O, and 0.12 g dm^−3^ KH_2_PO_4_ in distilled water, with the pH being adjusted with 0.1 M HCl. Lacrimal fluid of pH = 7.4 was prepared by dissolving 2.2 g dm^−3^ NaHCO_3_, 6.26 g dm^−3^ NaCl, 1.79 g dm^−3^ KCl, 96.4 mg dm^−^ MgCl_2_·6H_2_O, and 73.5 mg dm^−3^ CaCl_2_·H_2_O in distilled water, with the pH being adjusted with 1 M HCl. Mucin (porcine gastric mucin type II) was purchased from Sigma Aldrich. Mucin dispersions were prepared with simulated lacrimal fluid and stirred for 8 h. As reference system eye drop formulation from the market was used, consisting of dextran, hypromellose, benzalkonium chloride, EDTA, KCl, NaCl, and water for injection, with the pH being adjusted with HCl and NaOH. Sodium hyaluronate (HA) (MW: 4350 kDa) was purchased from RichterGedeon Ltd. (Budapest, Hungary).

### 2.2. Synthesis of Cationic ThioPASP-DME Polymers

**Figure 1:**
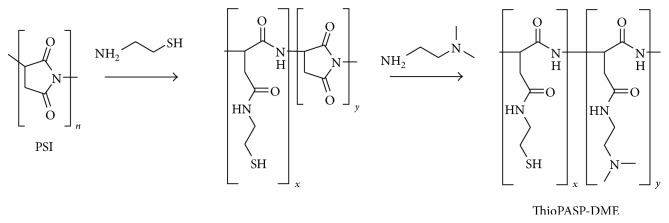
Synthesis of cationic ThioPASP-DME polymers.

The precursor polymer of cationic ThioPASP, polysuccinimide (PSI), was synthesized by the thermal polycondensation of L-aspartic acid in a solvent-free reaction at high temperature and reduced pressure. PSI and cysteamine were dissolved in DMF under a nitrogen atmosphere and the solution was stirred for 72 h at room temperature. An excess of* N*,*N*-dimethylethylenediamine was then added and the mixture was stirred for another 24 h under a nitrogen atmosphere. The polymer was precipitated in an excess of ethyl acetate and washed with ethyl acetate and acetone to yield the free base of poly[(*N*-mercaptoethylaspartamide)-co-(*N*-(*N*′,*N*′-dimethylaminoethyl)aspartamide)] (ThioPASP-DME). The polymers are abbreviated as ThioPASP-DME *X*, where *X* is the percentage molar ratio of the* N*-mercaptoethyl aspartamide to the total number of repeating units (Figure 1).

### 2.3. Ocular Compatibility Tests

Osmolality and pH were measured in 10% w/w aqueous solutions of ThioPASP-DME. Osmolality measurements based on the freezing point depression of a solution were carried out with an automatic osmometer (Knauer Semimicro Osmometer, Germany) in 3 parallels. 150 *μ*L of the solution in a test tube was placed into the instrument, and the sample was overcooled to a temperature lower than its freezing point. Mixing was next applied, which promoted crystallization of the sample. During the crystallization, the temperature automatically rose to the freezing point of the sample and remained at that temperature for a time. The osmolality (in mOsmol L^−1^) of the sample was calculated from the freezing point depression.

The pH of ThioPASP-DME solutions prepared with distilled water was determined with a pH meter (Testo 206-pH2, UK) [10].

### 2.4. Optical Tests

Optical tests were performed by the measurement of transmittance with a UV-spectrophotometer (Thermo Scientific Evolution 201 UV-Visible Spectrophotometer, Thermo Fischer Scientific, Shanghai, China) in the wavelength range 200–800 nm. In our investigations, the thickness of the samples was 10 mm. The transmittance in aqueous solutions of ThioPASP-DME was determined at 10% w/w.

The* refractive index* of the same solution was measured with an Abbe refractometer [10].

### 2.5. Wettability of Ocular Surfaces

The wettability of ocular surfaces with cationic ThioPASP formulations (10% w/w ThioPASP-DME polymers in PBS) was studied with an OCA Contact Angle System (Dataphysics OCA 20, Dataphysics Inc., GmbH, Germany). Microscopic slides were covered with 20 *μ*L cm^−2^ 5% w/w mucin dispersion in PBS and dried at room temperature for 24 h to model the ocular surface. Drops of ThioPASP-DME solutions were deposited on the surfaces. The degree of wetting was determined by measuring the contact angle by drop shape analysis. If the contact angle of the drops is <90°, the applied system will probably spread easily on the ocular surface, which can promote the interactions between the mucus layer and the formulation.

### 2.6. Rheology

The effect of the oxidative agent on the polymer solutions and the interaction between the polymer solution and the ocular mucin were investigated by rheology. The rheological properties were studied with a Physica MCR101 rheometer (Anton Paar, Austria). The measuring device was cone and plate type (the diameter was 25 mm, the gap height in the middle of the cone was 0.046 mm, and the cone angle was 1°). ThioPASP-DME was dissolved in PBS and the gelation test was initiated by the addition of model oxidant. For the investigation of the interaction between the polymer and the ocular mucin, the polymer was mixed with a mucin dispersion in PBS and in the presence of 20% w/w model oxidant (the final mucin concentration was 5% w/w, while the final polymer concentration was 10% w/w). As blank measurement, the polymer solution without mucin was measured. The structural changes in the formulation were characterized by frequency sweep tests. The oxidative effect on the eye can induce gelation, and the interactions between ThioPASP-DME and mucin can also result in structural changes; the storage modulus (*G*′) was therefore measured in two different rheological tests. *G*′ indicates the gel state and can also provide information on the strength of the interactions. The higher the value of *G*′, the stronger the gel structure formed. In the first rheological test, *G*′ was plotted for 20 min after the addition of model oxidant, using a strain of 1% and an angular frequency of 0.1 s^−1^ at 25°C. This test follows the possible gelation process. In the second rheological test, *G*′ was determined over the angular frequency range from 0.1 to 100 s^−1^, at a strain of 1% and at 25°C. This test provides information concerning the structure and the strength of the interactions [9].

### 2.7. Tensile Test

Tensile test also provides information on the interfacial interaction of the polymer and the ocular surface. Measurements were performed with a TA-XT Plus (Texture analyser (ENCO, Spinea, I)) instrument equipped with a 1 kg load cell and a cylinder probe with a diameter of 1 cm. The force and work needed to separate the polymer solution from the ocular surface are measured, which can characterize the strength of the interaction. Three different test conditions were used: the ocular surface was modelled (1) with 50 *μ*L of an 8% w/w mucin dispersion made with simulated lacrimal fluid (pH = 7.4) on a filter paper (*in vitro* condition), (2) with excised porcine conjunctiva (*ex vivo* condition), and (3) with simulated lacrimal fluid on a filter paper (as a blank measurement).

The porcine conjunctiva was obtained from a slaughterhouse, freshly detached from the connective tissue and stored at −20°C until the measurement. 10 parallel measurements were carried out. Test conditions were as follows: 20 *μ*L of the ThioPASP-DME (containing 20% w/w oxidant and 10% w/w polymer) and HA (0.5 and 1.0% w/w) solutions were attached to a cylinder probe and placed in contact with the test substrates (*in vitro*,* ex vivo*, and blank). A 2500 mN preload was used for 3 min to establish intimate contact between the sample and the test surface. The cylinder probe was then moved upwards to separate the sample from the substrate at a prefixed speed of 2.5 mm min^−1^. The work of adhesion (*A*, mN·mm) was calculated as the area under the force versus displacement curve (AUC) [9].

### 2.8. Statistical Analysis

The results were evaluated and analysed statistically with GraphPad Prism software (version 5). One-way and two-way ANOVA (with Bonferroni posttests) analysis were applied [11]. The values are expressed as means ± standard deviation (SD). A level of *p* ≤ 0.05 was taken as significant, *p* ≤ 0.01 as very significant, and *p* ≤ 0.001 as highly significant.

## 3. Results

### 3.1. Ocular Compatibility Tests

During ocular drug delivery formulation, several excipients are used which can change the physical and physiological properties of the ocular surface and the stability of the tear film [4, 12, 13]. The osmolality and the pH of the ThioPASP-DME solutions were therefore measured to determine the physicochemical properties of the solutions. The results are presented in Table 1.

Aqueous solutions of ThioPASP-DME polymers showed strong hypoosmolality (<100 mOsmol L^−1^), while the reference system was close to isotonic (301.4 mOsmol L^−1^). The solutions were alkaline (pH > 7). In order to modify the pH of the polymer solution close to that of the tear film (pH = 7.4), the synthesis was extended with a neutralization step. As a result, the pH of this polymer solution was approximately the physiological pH and that of the reference system (pH = 6.07), while the osmolality increased but remained hypoosmotic (<200 mOsmol L^−1^).

### 3.2. Optical Tests

**Figure 2:**
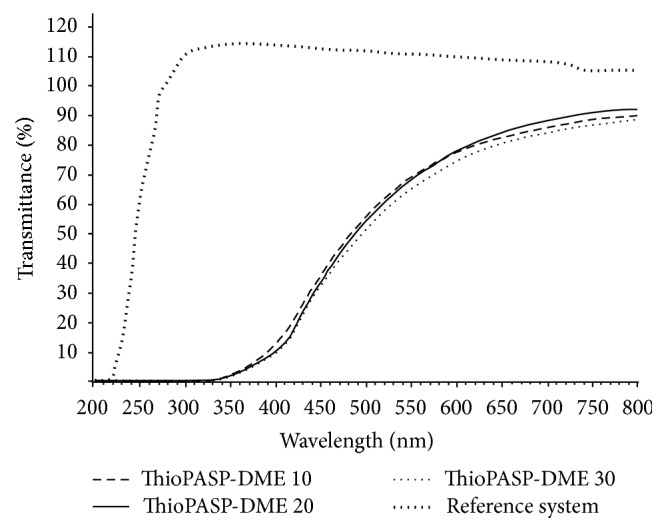
Transmittance of ThioPASP-DME solutions.

Transmittance spectra of 10% w/w ThioPASP-DME solutions were determined to study the effects of the solutions on the vision. The transmittance curves are depicted in Figure 2.

The ThioPASP-DME solutions are not colourless but slightly yellow, though the transmittance is high over almost the whole range of the visible spectrum. There was no significant effect of the degree of modification (composition) of the ThioPASP-DME. Interestingly, the polymer solutions exhibited a noteworthy UV cut-off at 350 nm; this behaviour can be favourable in the event of eyes exposed to heavy UV radiation.

The refractive indices of the ThioPASP−DME 10, 20, and 30 and the reference solutions were 1.3483, 1.3491, 1.3499, and 1.3350, respectively.

### 3.3. Wettability of the Ocular Surface

**Figure 3:**
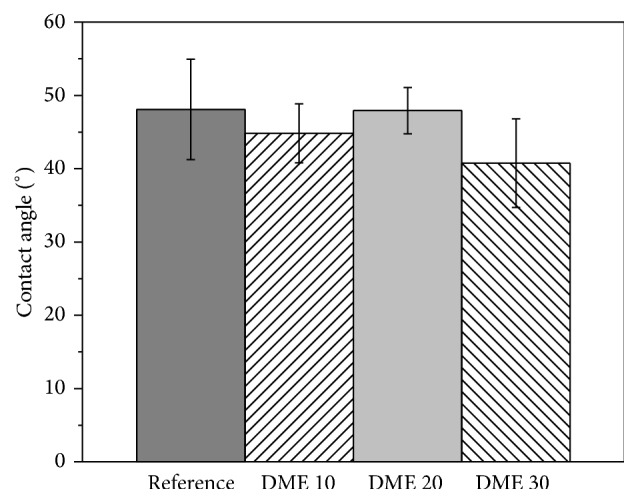
Contact angles of ThioPASP-DME solutions.

As it is intended to use the ThioPASP-DME solutions in liquid eye drops, their spreading on the ocular surface is an important aspect. In our tests, the ocular surface was modelled with a microscope slide covered with a mucin dispersion. The measured contact angles are to be seen in Figure 3.

The results indicate that the tested polymer compositions provide favourable wetting conditions on the model surface, because the contact angle is <90°.

#### 3.3.1. Rheology

The ThioPASP polymer solutions exhibited* in situ* gelling [9]; the gelation ability of ThioPASP-DME solutions was also tested. In the* in vitro* tests,* in vivo* factors that affected the gelling properties were applied, such as the model oxidant (as oxidative stress) and mucin (as a physiological component of tear film). The gelation (storage modulus (*G*′)) was first determined with and without mucin in the presence of the oxidant.

No gelation was observed in the case of ThioPASP-DME solutions. The *G*′ values did not increase during the examination time, which was in contrast with findings in our previous work, in which solutions of ThioPASP demonstrated abrupt increases in *G*′ within a few minutes. Even the addition of mucin did not induce gelation in the case of ThioPASP-DME.

**Figure 4:**
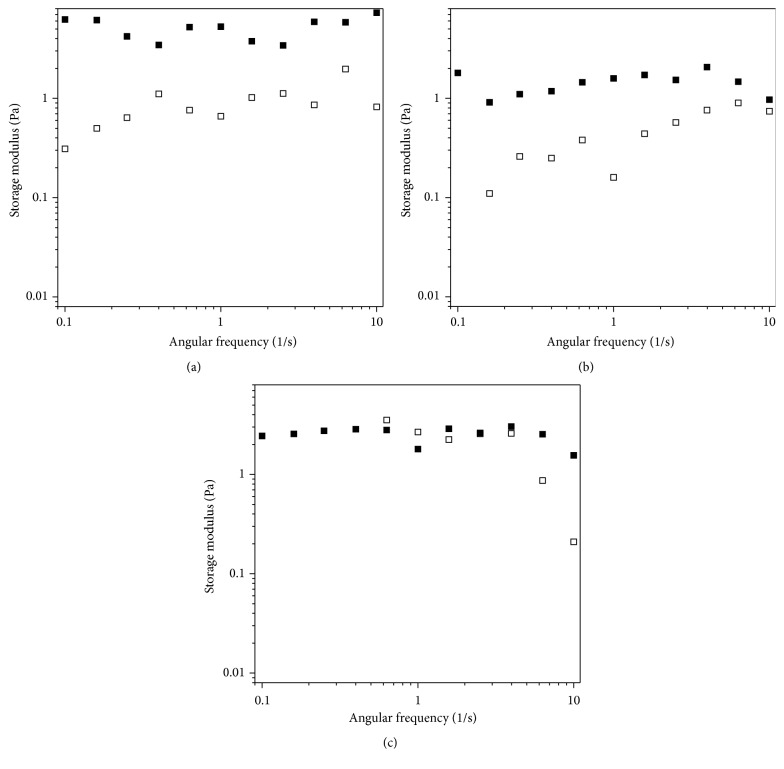
Frequency sweep tests of (a) ThioPASP-DME 10, (b) ThioPASP-DME 20, and (c) ThioPASP-DME 30 with (filled symbols) or without (open symbols) mucin.

Frequency sweep tests were performed with the aim of determining any synergetic interaction between the ThioPASP-DME and the mucin (Figure 4). This method is based on the determination of synergistic increases in rheological parameters (*G*′) after the sample is mixed with a mucin dispersion. The increase in *G*′ is caused by chemical and physical bond formation between the mucin and the polymer chains [9, 14–16].

A minor increase in *G*′ was observed in the presence of mucin for the lower degrees of modification, indicating the interaction of the polymer and the mucin. The modulus depended strongly on the angular frequency for the same compositions without mucin and the frequency dependence was slightly reduced in the presence of mucin, suggesting the formation of a weak network. These differences were not observed for the highest degree of modification (ThioPASP-DME 30), where a rather frequency-independent *G*′ was observed both with and without mucin.

#### 3.3.2. Tensile Test

**Figure 5:**
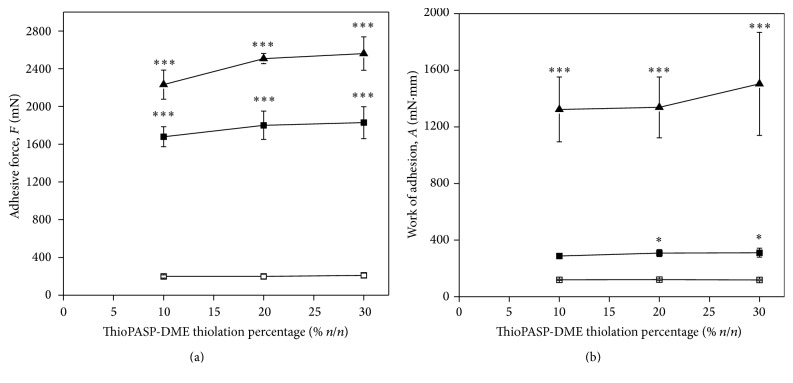
(a) Adhesive force and (b) work of adhesion of ThioPASP-DME solutions under (□) blank, (■)* in vitro,* and (▲)* ex vivo* conditions as functions of the polymer modification (^*∗*^ *p* ≤ 0.05, significant difference from the blank; and ^*∗∗∗*^ *p* ≤ 0.001, highly significant difference from the blank).

Force was measured as a function of displacement during tensile tests. The adhesive force (the maximum in the curve) and the work of adhesion (the AUC) were calculated [17]. The possible adhesion of ThioPASP-DME solutions to the ocular surface was determined through contacts with lacrimal fluid (blank), mucin dispersion (*in vitro*), and porcine eye conjunctiva (*ex vivo*). The adhesive force (*F*) and the work of adhesion (*A*) are shown in Figure 5.

Comparison of the blank with the* in vitro* and* ex vivo* results revealed significant increases in* F* and* A* (Figure 5), reflecting the interactions of the ThioPASP-DME polymer with the model surfaces. The highest values were observed in the case of the excised porcine conjunctiva, suggesting that the polymer interacts not merely with the mucin, but also with the other components of the ocular surface. The adhesive force and the work of adhesion values did not vary appreciably with the composition, but the substrate applied during the measurements affected these values strongly, as discussed below.

The mucoadhesivity of the new polymers was compared with that of hyaluronic acid solutions. HA as viscosity enhancing agent has been investigated for years as an active component of formulations applied in DED. Sodium hyaluronate increases the residence time and the precorneal tear film stability and the corneal wettability. It also decreases the evaporation rate of the tear film and improves the healing mechanisms of the cornea [18–21]. The generally applied concentration of HA in artificial tear is 0.1–0.5% w/w. In our work, mucoadhesion of 0.5 and 1.0% w/w HA solutions on porcine conjunctiva was measured and compared with that of 10% ThioPASP-DME 10 solution.

**Figure 6:**
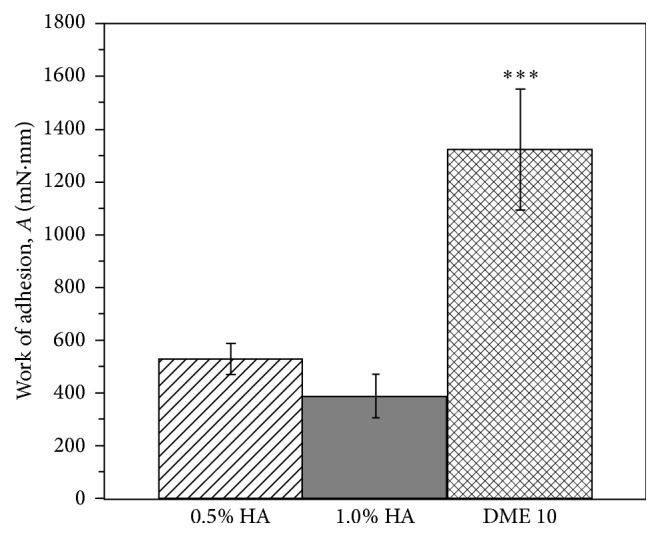
Work of adhesion of polymer solutions under* ex vivo* conditions (^*∗∗∗*^ *p* ≤ 0.001, highly significant difference from 0.5% and 1.0% w/w HA).

Under* ex vivo* condition, the ThioPASP-DME 10 displayed significantly higher mucoadhesivity compared with that of HAs (Figure 6). This phenomenon can be explained by the structure of the new cationic polymer. The elevated work of adhesion value may indicate the formation of disulfide bond and ionic interactions between the polymer chains and the ocular surface, while the viscosity of the polymer solution remained at a moderate level. Complex viscosity of the 0.5% HA, 1.0% w/w HA, and ThioPASP-DME solutions (at 10 Hz) were 80, 1680, and 580 mPas, respectively. Increase of the HA concentration from 0.5 to 1.0 did not affect the work of adhesion.

## 4. Discussion

DED is a multifunctional disease involving the tears and the ocular surface, associated with an increased osmolality of the tear film and inflammation of the ocular surface. The two most common causes of DED are insufficient tear production and excessive tear evaporation, both of which lead to hyperosmolality, ocular damage, or discomfort [3, 22]. Environmental factors (such as air dryness, pollution, or working close to a computer monitor) may increase a tear film dysfunction and cause further evaporative dry eye [23].

Because of the multifactorial pathology of DED, the therapy tends to be very varied. In the main treatments, artificial tears are used, especially preservative-free products, but unfortunately these provide only palliative therapy. In the event of inflammation, artificial tears are combined with oral omega-3 supplements, mucin secretagogues, short-term steroids, and daily cyclosporine A. When the DED is more severe, autologous serum, oral tetracyclines, prosthetic lenses, and systemic immune-suppressants are administered [2, 3]. Locally applied eye drops are used several times per day, which can cause toxic side effects because of the preservative (especially benzalkonium chloride) present in the formulations. These preservatives are cytotoxic to the ocular surface by modifying the lipid phase of the tear film [19].

Osmolality has been deeply investigated in DED and is considered to be a very important factor. The osmolality of the tears in a normal eye is 310 to 334 mOsm L^−1^, but in DED the osmolality is higher. One aim of artificial tears is to counter this hyperosmolality, but the effect is generally only temporary. The osmolality of artificial tears is usually in the interval from 181 to 354 mOsmol L^−1^ [24, 25].

In the treatment of DED, stabilization of the tear film is also very important. The tear film is stable for only a short time, because it ruptures in consequence of the concentration gradients and dispersion forces on the mucus layer. The rupture results in the loss of moisturization of the cornea, so that dry spots are formed, which irritate the corneal nerve endings and induce blinking. Thanks to the eyelid movements, a new tear film spreads over the eye surface. The dispersion forces, the interfacial tension, and the viscous resistance of the mucus layer affect the duration of rupture of the mucin layer and the breakup time of the tear film [4].

When all of these factors are taken into consideration, it appears clear that most of the physicochemical properties of the optimum eye drop formulation must be similar to those of the tear film and it must be hypoosmotic to balance the hyperosmotic tears in DED.

In this work, we synthetized and characterized ThioPASP-DME, cationic thiolated polyaspartamide bearing both cationic tertiary amine and redox-responsive thiol pendant groups, as a potentially mucoadhesive and tear film-stabilizing excipient in the therapy of DED. The aim was the synthesis of a mucin analogue polymer which can interact with the ocular mucin via disulphide linkages and the ionic interactions between the positively charged polymer and the negatively charged mucosal surface. Thanks to these complex interactions, a continuous polymer network is formed on the surface, thereby preserving the tear film with maintenance of the hydration of the ocular surface. We assume that ThioPASP-DME polymers can function as ophthalmic drug demulcents, defined in US Food and Drug Administration (FDA) monograph 21 CFR 349 as water-soluble polymers applied topically to protect and lubricate mucous membrane surfaces and to temper dryness and irritation.

We first investigated the physiological acceptability of our formulations. Eye lubricants are recommended to be neutral or slightly alkaline. The pH of the ThioPASP-DME polymer solutions (pH = 8.7–8.8) was higher than that of normal tears (pH = 7.4) and could be therefore adjusted by using hydrochloric acid.

The polymer solutions (10% w/w) were hypoosmotic (87–90 mOsmol L^−1^) allowing the addition of other components, which is favourable in the therapy of DED. The neutralization process resulted in lower pH but higher osmolality (183.67 ± 1.25 mOsmol L^−1^), which is in the range of the osmolality of artificial tears (from 181 to 354 mOsmol L^−1^) [24, 25], but this also allows the inclusion of further additives to the formulation. Ocular lubricants utilized in DED usually contain electrolytes (e.g., bicarbonate, potassium, and other electrolytes), surfactants, and various types of viscosity-increasing agents [26, 27].

Optical tests were performed in order to determine the degree of visual disturbance caused by these polymer solutions. The transmittance of the polymer solutions is slightly modified over a broad range of the visible spectrum and their refractive indices approximate to that of the tears. Thus, they do not greatly affect the quality of vision, while in addition they have a partial UV-filtering effect, which can be favourable in ophthalmic therapy.

The polymer solutions can readily spread on the simulated eye surface, as indicated by the low contact angles. This means that the formulations have the ability to establish strong interactions with the surface and to resist elimination immediately after administration.

The ThioPASP polymers are redox-sensitive and undergo gelling in response to oxidative stress or agents [9]. The present work revealed that the solutions of the ThioPASP-DME polymers did not form gels in response to an oxidative effect. This behaviour can be advantageous, because a sticky feeling and a foreign body sensation can be avoided and the swelling gel does not cause dehydration. On the other hand, ThioPASP-DME interacts with mucin, as indicated by the elevated *G*′ in rheological experiments, with the polymer therefore remaining on the surface without causing a noteworthy increase in viscosity.

Tensile tests likewise verified the good adhesion of the polymer solution to the ocular surface. Besides hydrogen bonds, thiolated polymers are able to form covalent bonds with the cysteine-rich subdomains of mucin. We additionally immobilized other side groups with cationic, positively charged groups, so that ionic interactions can also occur [4]. Changes in the degree of thiolation did not affect the adhesion appreciably, but an increased degree of thiolation is not recommended because a higher number of thiol side groups may result in lower stability of the polymers against atmospheric oxidation during storage. Oxidation during storage may lead to a lower dissolution rate prior to application. The strongest adhesion was measured on excised porcine conjunctiva, which suggests that not only do the mucin-polymer interactions (disulphide bonds) play a role in the adherence, but other secondary interactions may also develop, improving the efficacy of the formulation.

ThioPASP-DME polymers showed better mucoadhesion compared with conventionally used HAs in DED, while the viscosity of their solution was not elevated.

## 5. Conclusion

We successfully adjusted the properties of ThioPASP-DME (pH and osmolality) to the desired physiological levels thereby resulting in a possibility to decrease side effects such as irritation and dehydration. In consequence of their similar structure to that of mucin, ThioPASP-DME solutions also have the ability to stabilize the tear film. They can interact with the ocular mucin and provide strong adhesion, ensuring an improved residence time and prolonged hydration of the ocular surface. Further beneficial properties of the polymer solutions, such as good spreading on the ocular surface, marked transmittance, and a partial UV-filtering effect, can provide new possibilities in the therapy of DED.

## Figures and Tables

**Table 1:** Osmolality and pH of aqueous ThioPASP-DME solutions (10% w/w).

ThioPASP-DME degree of modification (% *n*/*n*)	Osmolality (mOsm/L) in water Mean ± SD	pH
10	87 ± 0	8.79
10^*∗*^	183.67 ± 1.25	6.07
20	90 ± 2.94	8.80
30	89.67 ± 0.47	8.77
Reference	282 ± 2.45	6.65

^*∗*^Neutralized with HCl.
